# Synthesis and crystal structure of *fac*-[3-bromo-6-(1*H*-pyrazol-1-yl-κ*N*^2^)pyridazine-κ*N*^1^]tri­carbonyl­chlorido­rhenium di­methyl­formamide monosolvate

**DOI:** 10.1107/S2056989026006249

**Published:** 2026-06-18

**Authors:** Duval Donoso, Nicolás Biget, Nancy Pizarro, Andrés Vega

**Affiliations:** a Departamento de Ciencias Químicas, Universidad Andrés Bello, Av. República 275, Santiago, Chile; b Departamento de Ciencias Químicas, Universidad Andrés Bello, Quillota 980, Viña del Mar, Chile; c Centro de Nanociencia y Nanotecnología, CEDENNA, Manuel Rodríguez Sur 415, Santiago, Chile; Venezuelan Institute of Scientific Research, Venezuela

**Keywords:** pyrazolyl-pyridazine, rhenium(I), tricarbonyl chloride, crystal structure

## Abstract

The crystal structure of the title rhenium(I) complex [ReCl(C_7_H_5_BrN_4_)(CO)_3_]·C_3_H_7_NO or *fac*-[(pypyrBr-κ^2^*N*,*N*)Re(CO)_3_Cl]·DMF [pypyrBr = 3-bromo-6-(1*H*-pyrazol-1-yl)pyridazine] is reported. The compound was synthesized by reacting **Re(CO)_5_Cl** with **pypyrBr** [3-bromo-6-(1*H*-pyrazol-1-yl)pyridazine] in toluene under reflux, yielding orange crystals upon purification and crystallization. Structural analysis reveals a distorted octa­hedral coordination environment around the Re^I^ center, comprising three facial carbonyl ligands, one chloride, and the bidentate **pypyrBr** ligand. The ligand exhibits near planarity with minimal torsional deviation, and the N—Re—N bite angle is 73.50 (12)°. The complex crystallizes as a 1:1 di­methyl­formamide solvate, consolidated by hydrogen bonding between DMF and the ligand. A Cambridge Structural Database search confirmed the novelty of this structure, as no prior reports exist for **pypyrBr** or its metal complexes. Spectroscopic characterization (^1^H NMR, ^13^C NMR, IR) and elemental analysis support the proposed structure. This work expands the family of rhenium(I) tricarbonyl complexes with pyrazolyl-pyridazine ligands, relevant for photophysical and coordination chemistry applications.

## Abstract 2

The reaction of **pypyrBr** [3-bromo-6-(1*H*-pyrazol-1-yl)pyridazine] with **Re(CO)_5_Cl** in refluxing toluene leads to ***fac*****-[(κ^2^-*N*,*N*-pypyrBr)Re(CO)_3_Cl]** in a 48% yield. Crystallization leads to a 1:1 di­methyl­formamide solvate, with hydrogen bonding between the dDMFmol­ecule and the pyrazolyl-pyridazine fragment on the octa­hedral **[(pypyrBr)Re(CO)_3_Cl]** mol­ecule.

## 1. Chemical context

Pyrazolyl-pyridazine derivatives are versatile multidentate and chelating ligands, which are appealing candidates for the synthesis of metal complexes for diverse applications. From the synthetic point of view, their synthesis and derivatization are relatively easy. They are also planar, with limited conformational flexibility, which diminishes non-radiative deactivation paths in photophysical applications (Pizarro *et al.*, 2018 ▸). Examples of structurally determined 6-1*H*-pyrazolyl-3-halopyridazine are limited to 3-chloro-6-(1*H*-pyrazol-1-yl)pyridazine (Ather *et al.*, 2010*a* ▸), 3-chloro-6-(3,5-dimethyl-1*H*-pyrazol-1-yl)pyridazine (Ather *et al.*, 2010*c* ▸), ethyl 5-amino-1-(6-chloro­pyridazin-3-yl)-1*H*-pyrazole-4-carboxyl­ate methyl (Ather *et al.*, 2010*b* ▸), 5-(2-((*t*-but­oxy­carbon­yl)amino)­eth­yl)-1-(6-chloro­pyridazin-3-yl)-1*H*-pyrazole-4-carboxyl­ate (Kralj *et al.*, 2009 ▸) and 3-chloro-6-(4-chloro-3,5-dimethyl-1*H*-pyrazol-1-yl)pyridazine but to the best of our knowledge no structural determination for 3-bromo-6-(1*H*-pyrazol-1-yl)pyridazine (**pypyrBr**).

Examples of 6-1*H*-pyrazolyl-3-halopyridazine coordinated to transition metals have been described previously: chloro-[3-chloro-6-(1*H*-pyrazol-1-yl)pyridazine]-[1,2,3,4,5,6-η^6^-1-meth­yl-4-(propan-2-yl)benzene]­ruthenium(II) tetra­fluoro­borate (Mambanda *et al.*, 2022 ▸), chloro-[3-chloro-6-(pyrazol-1-yl)pyridazine-*N,N*’]-(η^6^-*p*-cymene)ruthenium(II) hexa­fluoro­phosphate (Gupta *et al.*, 2009 ▸), di­aqua­bis­[3-chloro-6-(pyrazol-1-yl)pyridazine]copper(II) dinitrate (Blake *et al.*, 1998*b* ▸) and (μ_3_-oxo)[μ_2_-3-chloro-6-(pyrazol-1-yl)pyrazine]­penta­kis­(μ_2_-acetato)­bis­(pyridine)­triruthenium(II) hexa­fluoro­phosphate (Dai *et al.*, 2009 ▸). Examples of rhenium(I) tricarbonyl complexes are limited to **[(3-bromo-6-(1*****H*****-pyrazol-1-yl)pyridazine)Re(CO)_3_Br]** (Saldías *et al.*, 2019 ▸).

In the present paper, we report the synthesis and structure of **[(pypyrBr)Re(CO)_3_Cl]** [pypyrBr: 3-bromo-6-(1*H*-pyrazol-1-yl)pyridazine].
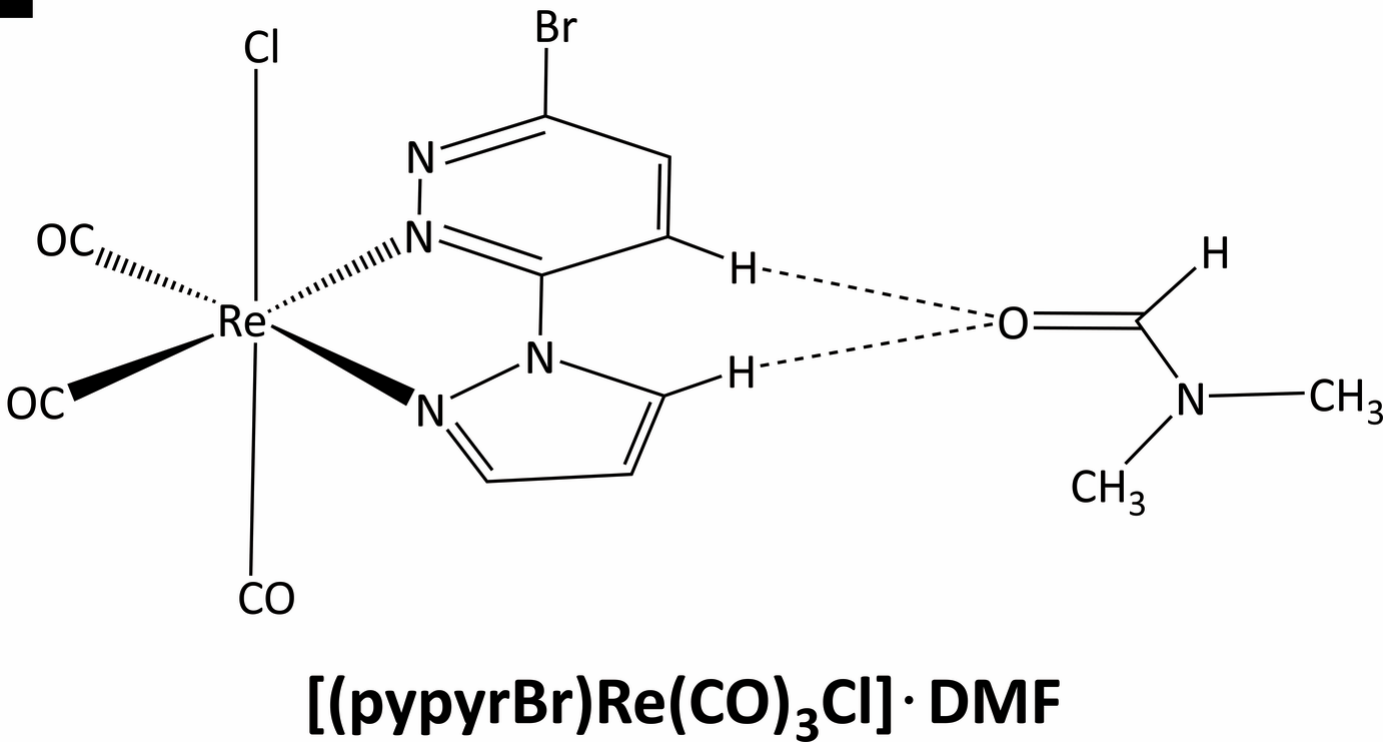


## 2. Structural commentary

**Figure 1:**
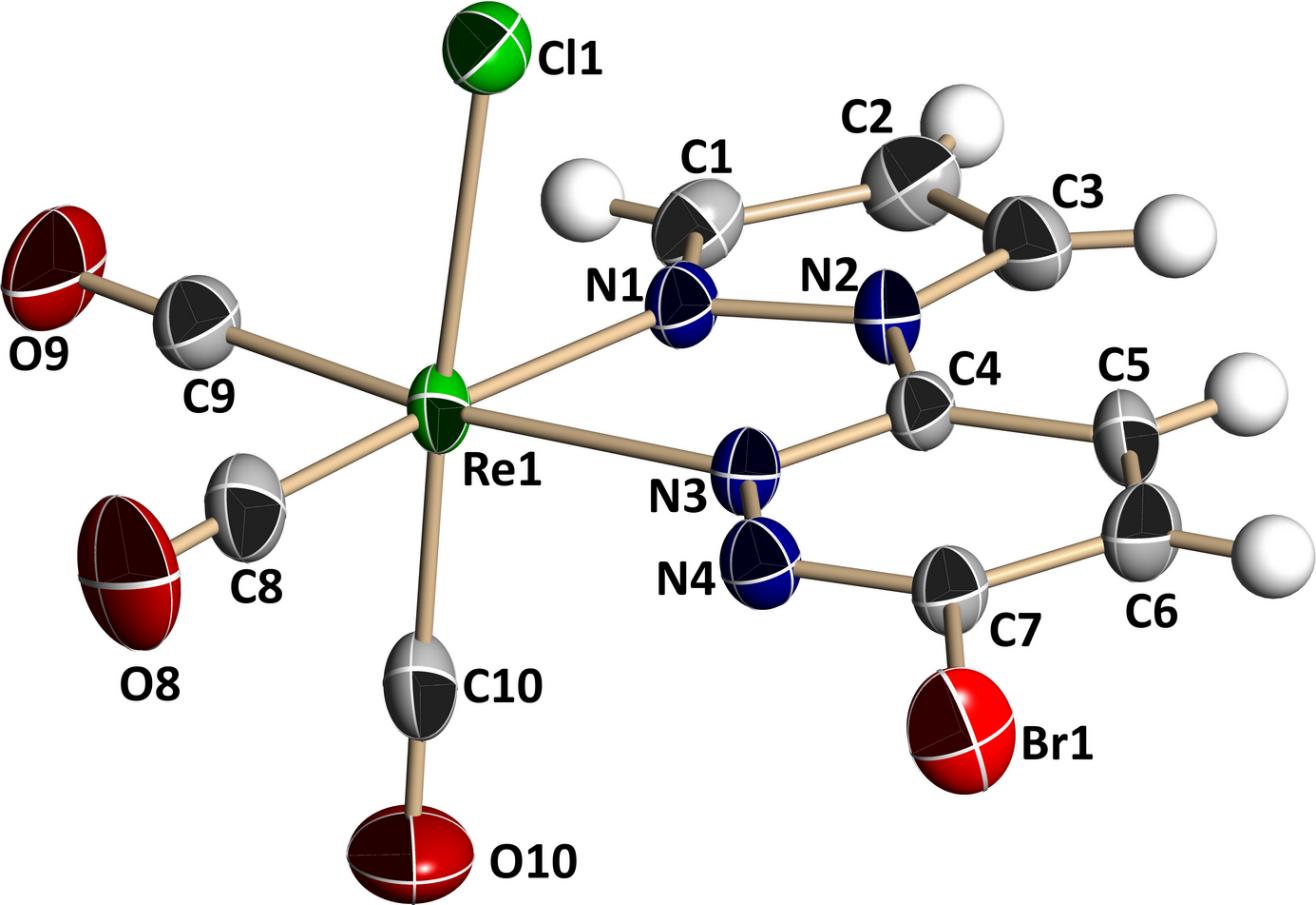
Mol­ecular view of the complex **[(pypyrBr)Re(CO)_3_Cl]** showing the partial numbering scheme. Atoms are shown as displacement ellipsoids at the 50% level of probability, except hydrogen, which are shown as arbitrary radii spheres. Solvating di­methyl­formamide mol­ecule omitted for the sake of clarity.

Fig. 1 ▸ shows a displacement ellipsoid plot of **[(pypyrBr)Re(CO)_3_Cl]**. The mol­ecule has a rhenium(I) centre, which displays a non-regular octa­hedron as coordination environment. The coordination number of six is completed by three carbonyl groups in a facial arrangement, a chloro ligand and a bidentate and chelating mol­ecule of **pypyrBr**. The **pypyrBr** ligand displays a highly planar and ***C*** conformation as required for coordination, which is reflected in the pyrazolyl-pyridazine torsion angles [N1—N2—C4—C5, 176.8 (3)°, C3—N2—C4—C5, 2.1 (6)°, C3—N2—C4—N3, −177.1 (4)° and N1—N2—C4—N3, −2.4 (5)°] and the rather low value of the biting N1—Re1—N3 angle [73.50 (12)°]. The dihedral angle between these two planar pyrazolyl and pyridazine groups is 4.0 (2)°.

## 3. Supra­molecular features

**Figure 2:**
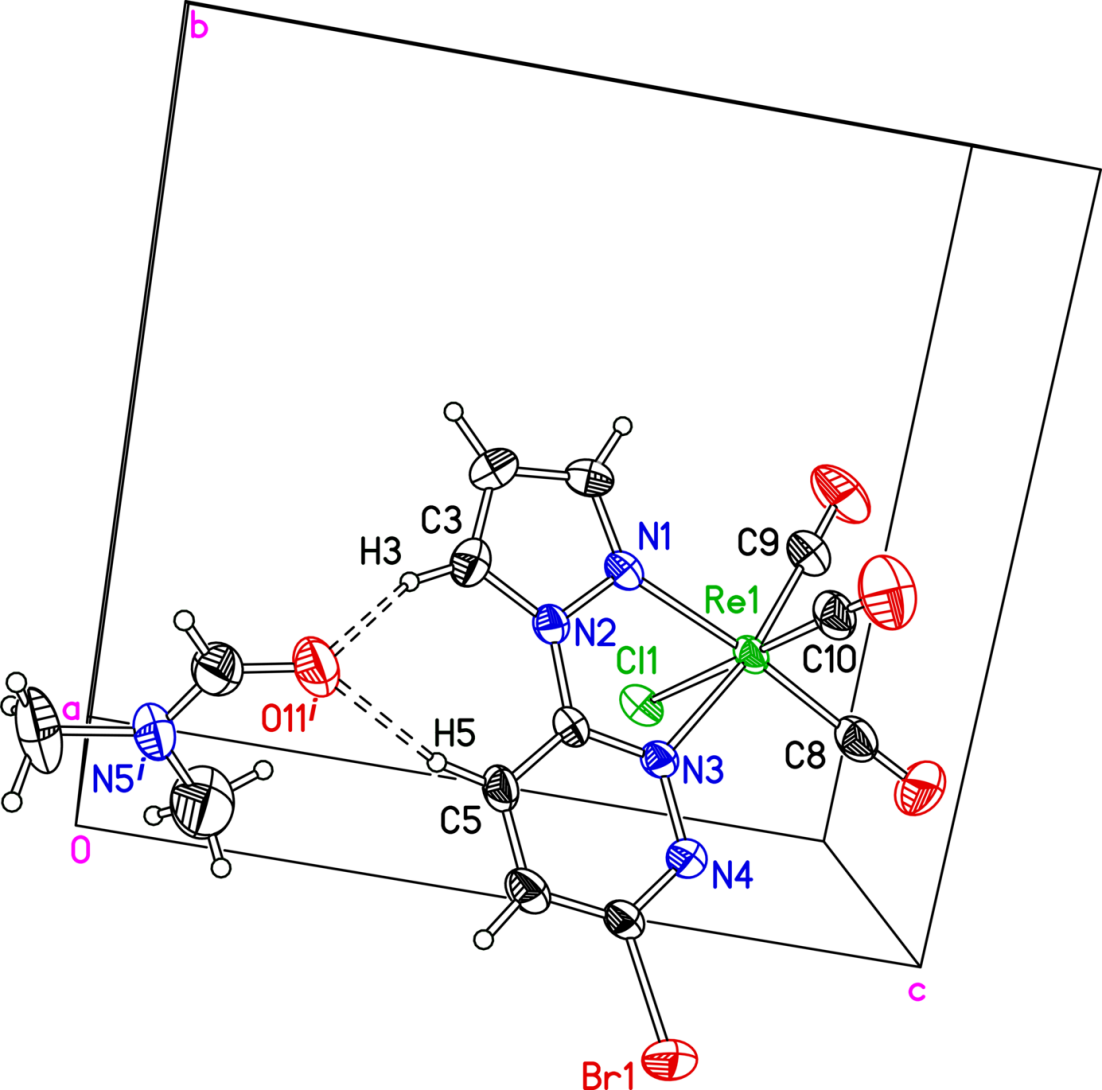
Mol­ecular view of the hydrogen bond between **[(pypyrBr)Re(CO)_3_Cl]** and solvating di­methyl­formamide.

As previously commented, the rhenium(I)tricarbonyl mol­ecule crystallizes as a 1:1 di­methyl­formamide solvate, with no evidence of partial occupancy. Fig. 2 ▸ shows how the carbonyl oxygen atom of the di­methyl­formamide mol­ecule accepts two hydrogen bonds to pyrazolyl, C3—H3, and pyridazine, C5—H5, with *D*⋯*A* = 3.251 (6) and 3.206 (5) Å respectively (Table 1 ▸).

## 4. Database survey

A search on the Cambridge Structural Database (v 6.00 updated to August 2025; Groom *et al.*, 2016 ▸) found no structural report for 3-bromo-6-(1*H*-pyrazol-1-yl)pyridazine or its transition-metal complexes.

The chloro analogue was reported as DUSZIB (Ather *et al.*, 2010*a* ▸). The 3,5-di­methyl­pyrazolyl analogue was reported as KUYZIO (Ather *et al.*, 2010*c* ▸). Transition-metal complexes of these two chloro analogues are found for copper(II) [COLPOJ and COLPUP (Li *et al.*, 2008 ▸), PUCYAN, PUCYER, PUCYIV, PUCYOB and PUCYUH (Blake *et al.*, 1998*b* ▸), QEPROS and QEPRUY (Blake *et al.*, 1998*a* ▸)], cobalt(II) (DOXPEM and DOXPIQ; An *et al.*, 2009 ▸), nickel(II) (DOXPOW; Li *et al.*, 2008 ▸), ruthenium(II/III) [FEWGOH, FEWGUN and FEWHAU (Mambanda *et al.*, 2022 ▸), IHEZEB and IHEZIF (Gupta *et al.*, 2009 ▸), WUCVAS (Dai *et al.*, 2009 ▸)] and rhodium(III) (MUSFOW; Gupta *et al.*, 2010 ▸).

## 5. Synthesis and crystallization

**pypyrBr**. A solution of 2.83 g (0.0416 mol) of pyrazole in tetra­hydro­furan (THF) was prepared under an inert atmosphere. To this solution, 0.2893 g (0.0416 mol) of lithium metal was added in a 1:1 molar ratio. The mixture was stirred at 343 K for approximately 3 h until the lithium was completely dissolved. The excess of unreacted lithium metal was then removed, and a solution of 9.917 g (0.0416 mol) of 3,6-di­bromo­pyridazine in THF was added dropwise. The reaction mixture was stirred at 343 K for 24 h, as shown in the reaction scheme. The resulting solid was washed with cold diethyl ether to yield the pure product **pypyrBr** (4.4238 g), corresponding to an approximate yield of 47%. Elemental analysis: calculated (%): C, 37.36; H, 2.24; N, 24.90. Found (%): C, 37.60; H, 2.43; N, 24.30. ^1^H NMR (400 MHz, CDCl_3_, δ ppm): 8.10 (*d*, *J* = 9.2 Hz, 1H, 5) and 7.75 (*d*, *J* = 9.2 Hz, 1H, 6) were assigned to the pyridazine protons, while the signals at 8.71 (*d*, *J* = 6.2 Hz, 1H, 3), 7.81 (*d*, *J* = 5.8 Hz, 1H, 1) , and 6.55 (*dd*, *J* = 2.7, 1.7 Hz, 1H, 2) correspond to the pyrazolyl fragment. ^13^C NMR (101 MHz, CDCl_3_, δ ppm): 154.00 and 145.13 ppm were assigned to pyridazine C7 and C4, bearing the bromo and pyrazolyl groups, respectively. The signals at 143.51 and 133.74 ppm correspond to the nitro­gen-bearing ring carbons C5 and C6. The resonances at 127.56, 119.74, and 109.36 ppm were attributed to the pyrazolyl carbons C3, C1, and C2, respectively. IR: ν_C–H_(arom): 3060, 3121, 3140, ν_C=N/C=C_(rings): 1570, 1520, 1455 and 1386 ν_C–Br_: 606.
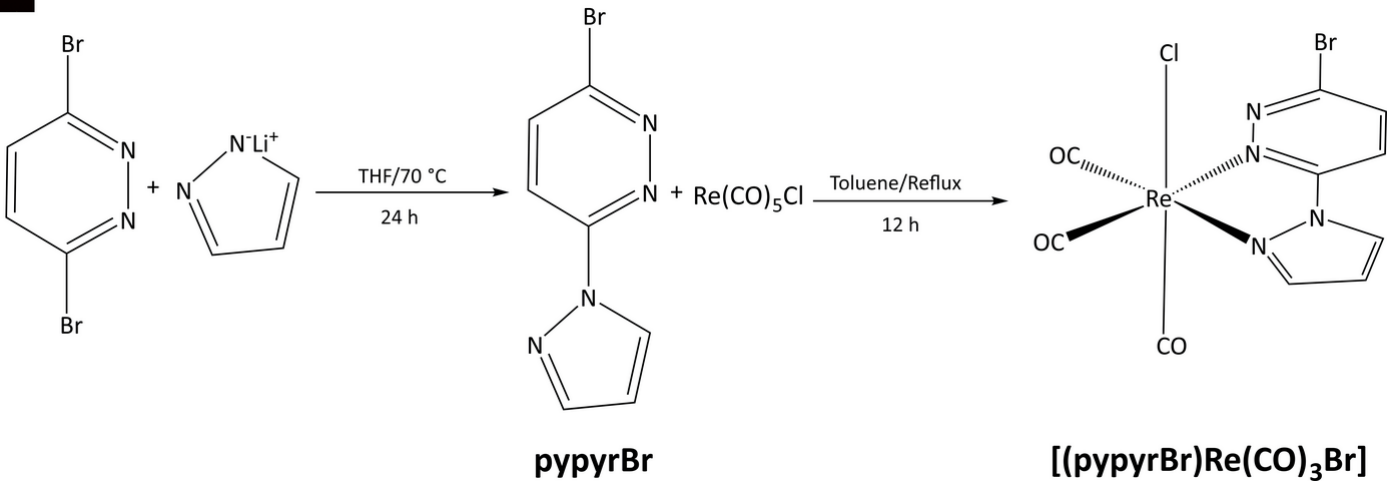


**[(pypyrBr)Re(CO)_3_Cl]**. The compound was synthesized by the reaction between penta­carbonyl­chloro­rhenium(I) and the ligand 3-bromo-6-(1*H*-pyrazol-1-yl)pyridazine (**pypyrBr**) in a 1:1 molar ratio, as shown in the reaction scheme. A solution of Re(CO)_5_Cl (500 mg, 1.38 mmol) in toluene was prepared in a round-bottom flask equipped for reflux. To this solution, **pypyrBr** (312.4 mg, 1.38 mmol), dissolved in toluene, was added dropwise under an inert atmosphere. The reaction mixture was refluxed for 12 h. After completion, the solvent was removed under reduced pressure. The crude product was purified by column chromatography using a mixture of di­chloro­methane/ethyl acetate (2:1) as the mobile phase, obtaining 350 mg of the product (approx. 48% yield). The pure product was crystallized from a THF/DMF (10:1) mixture, affording orange **[(pypyrBr)Re(CO)_3_Cl]·C_3_H_7_NO** crystals. Elemental analysis: calculated (%): C, 25.86; H, 2.00; N, 11.60. Found (%): C, 25.88; H, 2.04; N, 11.29%. The ^1^H NMR spectrum in DMSO-*d*_6_ displays the characteristic signals for the aromatic protons. The resonances for the pyridazine ring protons were assigned to the signals at δ 9.24 and 8.58 ppm. Regarding the pyrazole moiety, the protons H1 and H3 appear as doublets at δ 8.75 and 8.65 ppm, respectively, while the central proton H2 is observed at δ 7.04 ppm as a doublet of doublets. ^1^H NMR (400 MHz, DMSO-*d*_6_) δ 8.75 (*d*, *J* = 9.4 Hz, 1H, H-pyridazine 5), 9.24 (*d*, *J* = 3.1 Hz, 1H, H-pyrazole 3), 8.58 (*d*, *J* = 2.1 Hz, 1H, H-pyrazole 1), 8.65 (*d*, *J* = 9.3 Hz, 1H, H-pyridazine 6), 7.04 (*dd*, *J* = 3.1, 2.1 Hz, 1H, H-pyrazole 2). The ^13^C NMR spectrum in DMSO-*d*_6_ confirms the structure of the complex, displaying characteristic signals for the carbonyl ligands in a facial arrangement in the downfield region: a doublet at δ 196.68 (*J* = 33.5 Hz) corresponding to two coupled carbonyls, and a singlet at δ 189.57 ppm assigned to the in-plane carbonyl. Regarding the aromatic moiety, the pyridazine carbons bonded to the bromine atom (C7) and the pyrazole ring (C4) were assigned to δ 152.48 and 145.09 ppm, respectively, while the methine carbons of the same ring (C6/C5) appear at δ 147.36 and 137.29 ppm. Finally, the pyrazole resonances at δ 133.46 and 122.11 ppm were attributed to carbons C3 and C1, respectively, with the central carbon (C2) observed at δ 112.42 ppm. ^13^C NMR (101 MHz, DMSO-*d*_6_) δ 196.68 (*d*, *J* = 33.5 Hz, CO 8,9), 189.57 (*s*, CO 10), 152.48 (*s*, C-pyridazine 7), 147.36 (*s*, C-pyridazine 6), 145.09 (*s*, C-pyridazine 4), 137.29 (*s*, C-pyridazine 5), 133.46 (*s*, C-pyrazole 3), 122.11 (*s*, C-pyrazole 1), 112.42 (*s*, C-pyrazole 2). IR (cm^−1^): ν_C–H_(arom) 3090, 3030; ν_C_=_N_: 1670, 1580; ν_C≡ O_: 2025, 1940, 1905.

## 6. Refinement

Crystal data, data collection and structure refinement details are summarized in Table 2 ▸. Hydrogen atoms were placed in calculated positions (C—H: 0.93 Å for aromatic and C—H: 0.96 Å for aliphatic) and refined as riding [*U*_iso_(H) = 1.2 *U*_eq_(C) for aromatic and *U*_iso_(H) = 1.5 *U*_eq_(C) for aliphatic].

## Supplementary Material

Crystal structure: contains datablock(s) I. DOI: 10.1107/S2056989026006249/zn2046sup1.cif

Structure factors: contains datablock(s) I. DOI: 10.1107/S2056989026006249/zn2046Isup2.hkl

CCDC reference: 2562363

Additional supporting information:  crystallographic information; 3D view; checkCIF report

## Figures and Tables

**Table 1:** Hydrogen-bond geometry (Å, °)

*D*—H⋯*A*	*D*—H	H⋯*A*	*D*⋯*A*	*D*—H⋯*A*
C3—H3⋯O11^i^	0.93	2.41	3.251 (6)	150
C5—H5⋯O11^i^	0.93	2.33	3.206 (5)	158
C2—H2⋯Cl1^ii^	0.93	2.79	3.556 (4)	140
C6—H6⋯Cl1^iii^	0.93	2.75	3.614 (4)	154

Symmetry codes: (i) 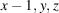; (ii) 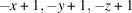; (iii) 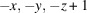.

**Table 2:** Experimental details

Crystal data	
Chemical formula	[ReCl(C_7_H_5_BrN_4_)(CO)_3_]·C_3_H_7_NO
*M*_r_	603.84
Crystal system, space group	Triclinic, *P* 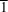
Temperature (K)	296
*a*, *b*, *c* (Å)	7.2454 (17), 11.136 (3), 11.426 (3)
α, β, γ (°)	90.941 (6), 93.728 (6), 102.141 (6)
*V* (Å^3^)	898.9 (4)
*Z*	2
Radiation type	Mo *K*α
μ (mm^−1^)	9.16
Crystal size (mm)	0.16 × 0.07 × 0.05

Data collection	
Diffractometer	Bruker CCD area detector
Absorption correction	Multi-scan (*SADABS*; Krause *et al.*, 2015 ▸)
*T*_min_, *T*_max_	0.199, 0.494
No. of measured, independent and observed [*I* > 2σ(*I*)] reflections	7009, 3512, 3277
*R*_int_	0.027
(sin θ/λ)_max_ (Å^−1^)	0.617

Refinement	
*R*[*F*^2^ > 2σ(*F*^2^)], *wR*(*F*^2^), *S*	0.023, 0.059, 1.04
No. of reflections	3512
No. of parameters	228
H-atom treatment	H-atom parameters constrained
Δρ_max_, Δρ_min_ (e Å^−3^)	1.14, −1.12

Computer programs: *APEX2* and *SAINT* (Bruker 2012 ▸), *SHELXL2014/7* (Sheldrick, 2015 ▸), *SHELXTL* (Sheldrick, 2008 ▸) and *publCIF* (Westrip, 2010 ▸).

## supplementary crystallographic information

### fac-[3-Bromo-6-(1H-pyrazol-1-yl-κN2)pyridazine-κN1]tricarbonylchloridorhenium dimethylformamide monosolvate Crystal data

**Table:**

[ReCl(C_7_H_5_BrN_4_)(CO)_3_]·C_3_H_7_NO	*Z* = 2
*M**_r_* = 603.84	*F*(000) = 568
Triclinic, *P*1	*D*_x_ = 2.231 Mg m^−^^3^
*a* = 7.2454 (17) Å	Mo *K*α radiation, λ = 0.71073 Å
*b* = 11.136 (3) Å	Cell parameters from 9860 reflections
*c* = 11.426 (3) Å	θ = 2.6–29.2°
α = 90.941 (6)°	µ = 9.16 mm^−^^1^
β = 93.728 (6)°	*T* = 296 K
γ = 102.141 (6)°	Prism, orange
*V* = 898.9 (4) Å^3^	0.16 × 0.07 × 0.05 mm

### fac-[3-Bromo-6-(1H-pyrazol-1-yl-κN2)pyridazine-κN1]tricarbonylchloridorhenium dimethylformamide monosolvate Data collection

**Table:**

Bruker CCD area detector diffractometer	3277 reflections with *I* > 2σ(*I*)
Radiation source: sealed tube	*R*_int_ = 0.027
phi and ω scans	θ_max_ = 26.0°, θ_min_ = 1.8°
Absorption correction: multi-scan (SADABS; Krause *et al.*, 2015)	*h* = −8→8
*T*_min_ = 0.199, *T*_max_ = 0.494	*k* = −13→13
7009 measured reflections	*l* = −14→14
3512 independent reflections	

### fac-[3-Bromo-6-(1H-pyrazol-1-yl-κN2)pyridazine-κN1]tricarbonylchloridorhenium dimethylformamide monosolvate Refinement

**Table:**

Refinement on *F*^2^	Primary atom site location: structure-invariant direct methods
Least-squares matrix: full	Secondary atom site location: difference Fourier map
*R*[*F*^2^ > 2σ(*F*^2^)] = 0.023	Hydrogen site location: inferred from neighbouring sites
*wR*(*F*^2^) = 0.059	H-atom parameters constrained
*S* = 1.04	*w* = 1/[σ^2^(*F*_o_^2^) + (0.0299*P*)^2^ + 0.4577*P*] where *P* = (*F*_o_^2^ + 2*F*_c_^2^)/3
3512 reflections	(Δ/σ)_max_ = 0.002
228 parameters	Δρ_max_ = 1.14 e Å^−^^3^
0 restraints	Δρ_min_ = −1.12 e Å^−^^3^

### fac-[3-Bromo-6-(1H-pyrazol-1-yl-κN2)pyridazine-κN1]tricarbonylchloridorhenium dimethylformamide monosolvate Special details

**Table:**

Geometry. All esds (except the esd in the dihedral angle between two l.s. planes) are estimated using the full covariance matrix. The cell esds are taken into account individually in the estimation of esds in distances, angles and torsion angles; correlations between esds in cell parameters are only used when they are defined by crystal symmetry. An approximate (isotropic) treatment of cell esds is used for estimating esds involving l.s. planes.Least-squares planes (x,y,z in crystal coordinates) and deviations from them (* indicates atom used to define plane)- 6.3159 (0.0076) x + 6.4497 (0.0188) y + 3.6505 (0.0219) z = 3.4009 (0.0136)* -0.0024 (0.0023) N1 * 0.0037 (0.0026) C1 * -0.0034 (0.0026) C2 * 0.0020 (0.0025) C3 * 0.0002 (0.0023) N2Rms deviation of fitted atoms = 0.0027- 6.0873 (0.0064) x + 7.0610 (0.0142) y + 3.5857 (0.0170) z = 3.6036 (0.0096)Angle to previous plane (with approximate esd) = The diher. 4.036 ( 0.230 )* 0.0055 (0.0024) N3 * -0.0044 (0.0026) C4 * 0.0004 (0.0028) C5 * 0.0023 (0.0029) C6 * -0.0012 (0.0028) C7 * -0.0025 (0.0025) N4Rms deviation of fitted atoms = 0.0032

### fac-[3-Bromo-6-(1H-pyrazol-1-yl-κN2)pyridazine-κN1]tricarbonylchloridorhenium dimethylformamide monosolvate Fractional atomic coordinates and isotropic or equivalent isotropic displacement parameters (Å^2^)

**Table:**

	*x*	*y*	*z*	*U*_iso_*/*U*_eq_	
Re1	0.24889 (2)	0.33339 (2)	0.76143 (2)	0.02623 (7)	
Br1	−0.32866 (7)	−0.11457 (4)	0.69106 (5)	0.05216 (14)	
Cl1	0.46364 (14)	0.21798 (9)	0.66737 (10)	0.0349 (2)	
N1	0.2276 (4)	0.4143 (3)	0.5929 (3)	0.0277 (7)	
N2	0.1065 (4)	0.3432 (3)	0.5096 (3)	0.0280 (7)	
N3	0.0312 (4)	0.2057 (3)	0.6545 (3)	0.0260 (7)	
N4	−0.0658 (5)	0.0996 (3)	0.6965 (3)	0.0308 (7)	
N5	0.7500 (5)	0.1716 (4)	0.0701 (3)	0.0429 (9)	
O8	0.2466 (6)	0.1981 (4)	0.9928 (3)	0.0656 (11)	
O9	0.5758 (5)	0.5317 (4)	0.8704 (4)	0.0637 (11)	
O10	−0.0322 (5)	0.4673 (4)	0.8641 (4)	0.0652 (11)	
O11	0.7352 (5)	0.2848 (3)	0.2338 (3)	0.0569 (9)	
C1	0.3002 (6)	0.5165 (4)	0.5393 (4)	0.0333 (9)	
H1	0.3870	0.5826	0.5750	0.040*	
C2	0.2290 (6)	0.5119 (4)	0.4225 (4)	0.0369 (10)	
H2	0.2597	0.5720	0.3673	0.044*	
C3	0.1068 (6)	0.4025 (4)	0.4057 (4)	0.0368 (10)	
H3	0.0360	0.3730	0.3365	0.044*	
C4	−0.0017 (5)	0.2319 (3)	0.5441 (3)	0.0253 (8)	
C5	−0.1346 (6)	0.1578 (4)	0.4659 (4)	0.0320 (9)	
H5	−0.1550	0.1797	0.3887	0.038*	
C6	−0.2336 (6)	0.0512 (4)	0.5084 (4)	0.0360 (9)	
H6	−0.3248	−0.0033	0.4616	0.043*	
C7	−0.1907 (5)	0.0282 (3)	0.6255 (4)	0.0294 (8)	
C8	0.2497 (6)	0.2478 (4)	0.9053 (4)	0.0394 (10)	
C9	0.4528 (6)	0.4573 (4)	0.8307 (4)	0.0374 (10)	
C10	0.0748 (6)	0.4186 (4)	0.8254 (4)	0.0385 (10)	
C11	0.7113 (7)	0.2651 (4)	0.1273 (5)	0.0474 (12)	
H11	0.6612	0.3217	0.0833	0.057*	
C12	0.8330 (11)	0.0826 (6)	0.1358 (6)	0.083 (2)	
H12A	0.9135	0.1237	0.2008	0.125*	
H12B	0.9061	0.0442	0.0854	0.125*	
H12C	0.7340	0.0211	0.1645	0.125*	
C13	0.7163 (9)	0.1547 (7)	−0.0550 (5)	0.0749 (19)	
H13A	0.6529	0.2163	−0.0853	0.112*	
H13B	0.6386	0.0747	−0.0734	0.112*	
H13C	0.8348	0.1616	−0.0901	0.112*	

### fac-[3-Bromo-6-(1H-pyrazol-1-yl-κN2)pyridazine-κN1]tricarbonylchloridorhenium dimethylformamide monosolvate Atomic displacement parameters (Å^2^)

**Table:**

	*U*^11^	*U*^22^	*U*^33^	*U*^12^	*U*^13^	*U*^23^
Re1	0.02852 (10)	0.02494 (10)	0.02296 (10)	0.00326 (6)	−0.00562 (6)	−0.00529 (6)
Br1	0.0578 (3)	0.0378 (3)	0.0503 (3)	−0.0140 (2)	0.0036 (2)	0.0034 (2)
Cl1	0.0346 (5)	0.0301 (5)	0.0399 (6)	0.0083 (4)	−0.0015 (4)	−0.0084 (4)
N1	0.0267 (16)	0.0262 (17)	0.0283 (19)	0.0026 (13)	−0.0024 (14)	−0.0057 (14)
N2	0.0315 (17)	0.0273 (16)	0.0226 (17)	0.0029 (13)	−0.0052 (13)	−0.0017 (13)
N3	0.0263 (16)	0.0243 (16)	0.0249 (18)	0.0014 (12)	−0.0034 (13)	−0.0016 (13)
N4	0.0336 (18)	0.0281 (17)	0.0285 (18)	0.0021 (14)	0.0005 (14)	0.0000 (14)
N5	0.048 (2)	0.049 (2)	0.031 (2)	0.0122 (18)	−0.0049 (17)	−0.0085 (18)
O8	0.093 (3)	0.062 (2)	0.036 (2)	0.006 (2)	−0.0076 (19)	0.0165 (18)
O9	0.043 (2)	0.060 (2)	0.078 (3)	−0.0043 (17)	−0.0128 (18)	−0.034 (2)
O10	0.055 (2)	0.085 (3)	0.065 (3)	0.037 (2)	0.0074 (19)	−0.013 (2)
O11	0.081 (3)	0.051 (2)	0.036 (2)	0.0139 (18)	−0.0114 (18)	−0.0101 (16)
C1	0.028 (2)	0.025 (2)	0.045 (3)	0.0029 (16)	−0.0007 (18)	−0.0006 (18)
C2	0.036 (2)	0.035 (2)	0.040 (3)	0.0069 (17)	0.0049 (19)	0.0120 (19)
C3	0.041 (2)	0.043 (2)	0.028 (2)	0.0114 (19)	0.0022 (18)	0.0054 (19)
C4	0.0251 (18)	0.0260 (18)	0.024 (2)	0.0055 (14)	−0.0019 (15)	−0.0056 (15)
C5	0.036 (2)	0.036 (2)	0.022 (2)	0.0058 (17)	−0.0064 (16)	−0.0049 (17)
C6	0.032 (2)	0.035 (2)	0.037 (3)	−0.0005 (17)	−0.0049 (18)	−0.0094 (18)
C7	0.030 (2)	0.0260 (19)	0.030 (2)	0.0014 (15)	−0.0018 (16)	−0.0052 (16)
C8	0.043 (2)	0.037 (2)	0.035 (3)	0.0051 (18)	−0.0069 (19)	−0.008 (2)
C9	0.035 (2)	0.037 (2)	0.039 (3)	0.0074 (18)	−0.0020 (19)	−0.0091 (19)
C10	0.042 (2)	0.040 (2)	0.030 (2)	0.0060 (19)	−0.0092 (19)	−0.0072 (19)
C11	0.057 (3)	0.043 (3)	0.042 (3)	0.012 (2)	−0.007 (2)	0.001 (2)
C12	0.125 (6)	0.071 (4)	0.070 (5)	0.056 (4)	0.012 (4)	0.000 (3)
C13	0.063 (4)	0.116 (6)	0.042 (3)	0.014 (4)	−0.001 (3)	−0.033 (3)

### fac-[3-Bromo-6-(1H-pyrazol-1-yl-κN2)pyridazine-κN1]tricarbonylchloridorhenium dimethylformamide monosolvate Geometric parameters (Å, º)

**Table:**

Re1—C10	1.904 (5)	O10—C10	1.143 (6)
Re1—C8	1.914 (5)	O11—C11	1.227 (6)
Re1—C9	1.915 (4)	C1—C2	1.396 (6)
Re1—N1	2.150 (3)	C2—C3	1.350 (6)
Re1—N3	2.180 (3)	C4—C5	1.392 (5)
Re1—Cl1	2.4982 (11)	C5—C6	1.366 (6)
Br1—C7	1.886 (4)	C6—C7	1.394 (6)
N1—C1	1.327 (5)	C1—H1	0.9300
N1—N2	1.370 (5)	C2—H2	0.9300
N2—C3	1.368 (5)	C3—H3	0.9300
N2—C4	1.397 (5)	C5—H5	0.9300
N3—C4	1.318 (5)	C6—H6	0.9300
N3—N4	1.354 (4)	C11—H11	0.9300
N4—C7	1.301 (5)	C12—H12A	0.9600
N5—C11	1.309 (6)	C12—H12B	0.9600
N5—C13	1.437 (7)	C12—H12C	0.9600
N5—C12	1.458 (7)	C13—H13A	0.9600
O8—C8	1.149 (6)	C13—H13B	0.9600
O9—C9	1.145 (5)	C13—H13C	0.9600
			
C10—Re1—C8	87.5 (2)	C6—C5—C4	116.6 (4)
C10—Re1—C9	89.04 (19)	C5—C6—C7	116.5 (4)
C8—Re1—C9	88.32 (19)	N4—C7—C6	125.5 (4)
C10—Re1—N1	93.10 (16)	N4—C7—Br1	115.9 (3)
C8—Re1—N1	174.39 (15)	C6—C7—Br1	118.6 (3)
C9—Re1—N1	97.27 (16)	O8—C8—Re1	178.1 (5)
C10—Re1—N3	94.22 (16)	O9—C9—Re1	178.9 (4)
C8—Re1—N3	100.89 (16)	O10—C10—Re1	178.5 (4)
C9—Re1—N3	170.35 (15)	O11—C11—N5	125.9 (5)
N1—Re1—N3	73.50 (12)	N1—C1—H1	124.5
C10—Re1—Cl1	176.65 (13)	C2—C1—H1	124.5
C8—Re1—Cl1	94.41 (14)	C3—C2—H2	126.9
C9—Re1—Cl1	93.74 (13)	C1—C2—H2	126.9
N1—Re1—Cl1	84.70 (9)	C2—C3—N2	107.2 (4)
N3—Re1—Cl1	82.74 (9)	C2—C3—H3	126.4
C1—N1—N2	105.2 (3)	N2—C3—H3	126.4
C1—N1—Re1	139.4 (3)	C6—C5—H5	121.7
N2—N1—Re1	115.4 (2)	C4—C5—H5	121.7
C3—N2—N1	110.4 (3)	C5—C6—H6	121.7
C3—N2—C4	131.5 (4)	C7—C6—H6	121.7
N1—N2—C4	117.9 (3)	O11—C11—H11	117.0
C4—N3—N4	119.4 (3)	N5—C11—H11	117.0
C4—N3—Re1	118.1 (2)	N5—C12—H12A	109.5
N4—N3—Re1	122.4 (2)	N5—C12—H12B	109.5
C7—N4—N3	117.9 (3)	H12A—C12—H12B	109.5
C11—N5—C13	122.5 (5)	N5—C12—H12C	109.5
C11—N5—C12	118.6 (4)	H12A—C12—H12C	109.5
C13—N5—C12	118.9 (5)	H12B—C12—H12C	109.5
N1—C1—C2	111.1 (4)	N5—C13—H13A	109.5
C3—C2—C1	106.1 (4)	N5—C13—H13B	109.5
C2—C3—N2	107.2 (4)	H13A—C13—H13B	109.5
N3—C4—C5	124.1 (3)	N5—C13—H13C	109.5
N3—C4—N2	114.8 (3)	H13A—C13—H13C	109.5
C5—C4—N2	121.1 (3)	H13B—C13—H13C	109.5
			
C1—N1—N2—C3	0.3 (4)	Re1—N3—C4—N2	−1.8 (4)
Re1—N1—N2—C3	−178.9 (3)	C3—N2—C4—N3	−177.1 (4)
C1—N1—N2—C4	−175.5 (3)	N1—N2—C4—N3	−2.4 (5)
Re1—N1—N2—C4	5.4 (4)	C3—N2—C4—C5	2.1 (6)
C4—N3—N4—C7	−0.9 (5)	N1—N2—C4—C5	176.8 (3)
Re1—N3—N4—C7	−178.8 (3)	N3—C4—C5—C6	−0.7 (6)
N2—N1—C1—C2	−0.6 (5)	N2—C4—C5—C6	−179.7 (4)
Re1—N1—C1—C2	178.2 (3)	C4—C5—C6—C7	0.0 (6)
N1—C1—C2—C3	0.7 (5)	N3—N4—C7—C6	0.3 (6)
C1—C2—C3—N2	−0.5 (5)	N3—N4—C7—Br1	−177.3 (3)
N1—N2—C3—C2	0.2 (5)	C5—C6—C7—N4	0.2 (7)
C4—N2—C3—C2	175.2 (4)	C5—C6—C7—Br1	177.8 (3)
N4—N3—C4—C5	1.2 (6)	C13—N5—C11—O11	179.5 (5)
Re1—N3—C4—C5	179.1 (3)	C12—N5—C11—O11	−1.5 (8)
N4—N3—C4—N2	−179.7 (3)		

### fac-[3-Bromo-6-(1H-pyrazol-1-yl-κN2)pyridazine-κN1]tricarbonylchloridorhenium dimethylformamide monosolvate Hydrogen-bond geometry (Å, º)

**Table:**

*D*—H···*A*	*D*—H	H···*A*	*D*···*A*	*D*—H···*A*
C3—H3···O11^i^	0.93	2.41	3.251 (6)	150
C5—H5···O11^i^	0.93	2.33	3.206 (5)	158
C2—H2···Cl1^ii^	0.93	2.79	3.556 (4)	140
C6—H6···Cl1^iii^	0.93	2.75	3.614 (4)	154

Symmetry codes: (i) *x*−1, *y*, *z*; (ii) −*x*+1, −*y*+1, −*z*+1; (iii) −*x*, −*y*, −*z*+1.
